# Development and implementation of a new part-time continuing education course in integrative oncology

**DOI:** 10.3205/zma001719

**Published:** 2024-11-15

**Authors:** Sarah Salomo, Jutta Hübner

**Affiliations:** 1 Universitätsklinikum Jena, Klinik für Innere Medizin II, Abteilung für Hämatologie und Internistische Onkologie, Jena, Germany

**Keywords:** education, integrative oncology, evidencebased medicine

## Abstract

**Introduction:**

Integrative oncology combines evidence-based methods of oncological therapy, supportive medicine, nutrition and physical activity as well as complementary medicine and can significantly improve the effectiveness of therapy and the quality of life for cancer patients. However, scientifically based continuing education in this area has so far rarely been available.

**Project outline:**

The part-time continuing education program in “Integrative Onkologie” at the University of Jena is the first in Germany to offer scientifically based training for various healthcare professions. The focus lies on evidence-based content regarding the diagnosis, therapy, prevention and survivorship of cancer from the fields of complementary medicine, sport and nutrition. The course comprises 3 semesters of distance learning with one attendance weekend in Jena and concludes with a Master’s thesis (M.Sc.). All healthcare professionals with a first university degree in medicine or health sciences and at least one year of professional experience are eligible for admission. The development of the study program was supported by surveys and statements from students and experts.

**Results:**

A demand survey of students (N=128) and statements (N=15) from healthcare professionals show that the course closes a gap in education and training. The course was seen as an attractive and suitable alternative to subject-specific consecutive Master's courses. Its interdisciplinary focus and its high relevance with regard to improvements in healthcare were rated particularly positive. From the students’ point of view, the topics of nutrition and physical activity were seen as particularly interesting, while the expert’s statements emphasized the need for evidence-based discussion, especially in the field of complementary medicine.

**Discussion:**

The course fills an important gap and provides evidence-based further training in integrative oncology. The presented conditions are rated as appropriate and the extended professional options and improvement of care in everyday practice are emphasized. The course was approved in April 2023 and has already started with the first cohort in the winter semester 2023 (October).

**Conclusion:**

The postgraduate course in integrative oncology at the University of Jena offers thorough training for healthcare professionals and fulfills the requirements for continuing education programs. It can therefore make a significant contribution to medical consultation needs and improving care in oncology and enable a faster path to specialized continuing education for many specialties.

## Zusammenfassung

**Einleitung:**

Die Integrative Onkologie vereint evidenzbasierte Methoden der onkologischen Therapie, supportiven Medizin, Ernährung und körperlichen Aktivität sowie komplementären Medizin und kann die Effektivität der Therapie sowie Lebensqualität krebserkrankter Personen deutlich verbessern. Allerdings ist eine wissenschaftlich fundierte Weiterbildung in diesem Bereich bisher kaum möglich.

**Projektbeschreibung:**

Das berufsbegleitende Weiterbildungsstudium „Integrative Onkologie“ an der Universität Jena bietet erstmals in Deutschland eine wissenschaftlich fundierte Weiterbildung für verschiedene Gesundheitsberufe. Der Fokus liegt auf evidenzbasierten Inhalten zur Diagnostik, Therapie, Prävention und Survivorship von Krebserkrankungen aus den Bereichen komplementäre Medizin, Sport und Ernährung. Der Studiengang umfasst 3 Semester Fernstudium mit einem Präsenzwochenende in Jena und schließt mit einer Masterarbeit (M.Sc.) ab. Zugelassen sind alle Berufstätigen des Gesundheitssystems mit einem ersten Hochschulabschluss in Medizin oder Gesundheitswissenschaften und mindestens einem Jahr Berufserfahrung. Die Entwicklung des Studiengangs wurde durch Umfragen und Stellungnahmen von Studierenden und Fachpersonen unterstützt.

**Ergebnisse:**

Aus einer Bedarfsumfrage unter Studierenden (N=128) sowie aus Stellungnahmen (N=15) von Fachpersonen des Gesundheitssektors wird deutlich, dass der Studiengang eine bisherige Lücke in der Aus- und Weiterbildung schließt. Das Studienangebot wurde als attraktive und gut geeignete Alternative zu fachspezifischen konsekutiven Masterstudiengängen gesehen. Besonders positiv wurde die interdisziplinäre Ausrichtung bewertet, sowie die hohe Relevanz für eine Versorgungsverbesserung. Aus Sicht der Studierenden werden die Themen Ernährung und Körperliche Aktivität als besonders interessant eingeschätzt, während die Stellungnahmen die Notwendigkeit der evidenzbasierten Auseinandersetzung insbesondere im Bereich der Komplementärmedizin unterstreichen.

**Diskussion:**

Der Studiengang füllt eine wichtige Lücke und bietet eine evidenzbasierte Weiterbildung in Integrativer Onkologie. Die vorgestellten Rahmenbedingungen werden als angemessen beurteilt und die erweiterte Berufsperspektive sowie Versorgungsverbesserung im Praxisalltag hervorgehoben. Der Studiengang wurde im April 2023 genehmigt und konnte bereits zum Wintersemester 2023 (Oktober) mit dem ersten Jahrgang starten.

**Schlussfolgerung:**

Das Weiterbildungsstudium in Integrativer Onkologie an der Universität Jena bietet eine fundierte Ausbildung für Gesundheitsberufe und erfüllt die Anforderungen an berufsbegleitende Weiterbildungen. Es kann damit einen wesentlichen Beitrag zum Beratungsbedarf und der Versorgungsverbesserung in der Onkologie beitragen und für viele Fachrichtungen einen schnelleren Weg in die spezialisierte Weiterbildung ermöglichen.

## 1. Introduction

Integrative oncology combines evidence-based treatments and methods from the fields of nutrition, physical activity and complementary medicine (e.g. phytotherapy) with conventional medical treatment. Particularly in the field of complementary medicine, a critical examination of the findings of evidence-based medicine is needed in order to provide not only the necessary specialist knowledge but also to address the political and social aspects in everyday healthcare (e.g. homeopathy, [1]). The topic is becoming increasingly important from the perspective of both the patients and the professions involved, as increasing life expectancy due to better therapies means that the treatment of oncological diseases is no longer just a question of cure, but of quality of life [2], [3]. However, integrative oncology, and complementary medicine in particular, has so far only been adequately implemented in very few further training courses [4] and is often only insufficiently addressed in undergraduate medical studies. This is illustrated in a study by Trimborn et al. (2013), in which around 73% of medical staff did not feel sufficiently informed on the subject of complementary medicine [5]. At the same time, a survey in the USA showed that 80% of doctors and 68% of nurses consider complementary medicine to be a support for cancer patients in dealing with the disease [6]. The first steps towards the systematic inclusion of integrative oncology in standard healthcare have already been taken with the S3 guideline “Komplementärmedizin in der onkologischen Behandlung” (Complementary Medicine in Oncological Treatment) introduced in June 2021. It recommends not only early and regular counseling of cancer patients regarding complementary medicine, but even calls for an obligation to do so [7]. However, in order to be able to advise and treat patients in a competent and layperson-oriented manner in standard care, specialized further training with an evidence-based foundation is required, which has so far rarely been available in Germany.

## 2. Project outline

The part-time and postgraduate course “Integrative Onkologie” at Friedrich Schiller University Jena (Faculty of Medicine and Faculty of Biosciences) and University Hospital Jena is the first postgraduate course in Germany for people from different healthcare professions with an academic background that aims to provide scientifically based knowledge and strengthen skills in the field of integrative oncology. Admission criteria include a first university degree in the field of medicine or health sciences (e.g. psychology, pharmacy, nursing, nutrition, sports science, occupational therapy, physiotherapy) and at least one year of professional experience in the health sector in terms of counseling, treatment or diagnostics of oncology patients or research-oriented activities (e.g. implementation of intervention projects or surveys) in the field of oncology. The course is based on level 7 of the “Deutscher Qualifikationsrahmen” *(German Qualifications Framework)* [8], [9], the guidelines of the “Kultusministerkonferenz” *(Conference of Ministers of Education and Cultural Affairs)* [10], as well as the overall revision to the “Muster-Weiterbildungsordnung” *(Model Continuing Education Regulations)* for doctors of the German Medical Association [11], including the subsequent amendment by the Thuringian Medical Association [12]. To ensure a target-group-oriented conceptualization a survey was conducted among students and statements were requested from several experts in the participating subject areas, who were asked in particular to determine the demand and conditions for the current study program. 

### 2.1. Structure

The online course spans over 3 semesters (60 ECTS in total) and can be completed entirely by distance learning, with the exception of one weekend of attendance at the end of the third semester. It concludes with a master’s thesis, which is usually written in the 3^rd^ semester, and awards the master of science degree. The content is taught in alternating synchronous online seminars (2 online seminars every 3 weeks, 3 hours each, starting at 6 p.m.) and asynchronously through exercises and covers a weekly workload of around 10 hours. The assignments include group work on the current research findings of the topics covered by the modules, as well as resulting strategies and recommendations for everyday practice, which are then discussed in class. This temporal and digital design of the course is intended to ensure the compatibility of studies, career and family, while at the same time ensuring the guided exchange of perspectives and professional discussion. The course covers 4 modules, which are taken in the first two semesters (2 modules per semester, 10 ECTS credits each, one examination each): 

#### 2.1.1. Complementary medicine

The module covers biologically based methods (e.g. nutritional supplements, phytotherapeutics), mind-body methods (e.g. yoga, tai chi), holistic systems (e.g. homeopathy, traditional Chinese medicine, anthroposophic medicine) and body-oriented methods (e.g. physiotherapy, manual medicine, cold and heat applications) as well as areas of energy medicine (e.g. acupuncture, aura therapy). A particular focus is placed on the critical examination and differentiation of alternative medicine from complementary medicine. All topics are analyzed and reviewed in terms of evidence-based medicine, while also discussing the possibilities and limitations of implementation in the overall oncological concept. 

#### 2.1.2. Nutritional medicine

The module provides in-depth knowledge of nutrition in the context of the overall oncological concept. Among other things, the module covers the basics of physiology, pathophysiology and biochemical aspects of nutrition (vitamins, trace elements, protein metabolism) as well as determining variables of the energy metabolism and its specifics in oncology. The benefits and risks of certain diets (vegetarian, vegan) and cancer diets are also discussed. The module provides knowledge on the role of nutrition in prevention and rehabilitation, as well as during oncological therapy and in certain clinical scenarios. 

#### 2.1.3. Physical exercise

The module provides enhanced knowledge and skills on various aspects of physical activity. Students analyze possible (contra-)indications of physical activity in certain oncological therapy and clincal scenariosi. Students learn about physical activity and sport with cancer in primary and tertiary prevention (history, consequences of inactivity) and symptom-specific influences. Selected training methods (oncological training therapy) are analyzed and discussed. 

#### 2.1.4. Integrative oncology

This module provides interdisciplinary knowledge about integrative oncology. The benefits and risks of symptomatic treatment options and their appropriateness in the context of integrative oncology are analyzed. A particular focus is placed on integrating the topics into lay communication and information for people with cancer and their relatives in order to strengthen resilience and self-efficacy. Students learn how to provide tailored advice on the benefits and risks of methods of complementary medicine, nutritional medicine and sports medicine, as well as the associated communication with lay people, other professional groups and the media. In addition, the ethical evaluation and legal principles of decision-making are discussed in the context of oncology.

## 3. Results

The online survey was sent to students in the health disciplines at Jena’s universities via the mailing lists of a total of 13 student councils, took an average of 10 minutes to complete and contained closed questions (Likert scale from 1-4) and open text responses. The survey was intended to assess personal opinions on content and general conditions as well as student demand.

In addition to demographic information, experience in the field of oncology (3 questions, e.g. “How would you rate your previous knowledge of integrative oncology and complementary medicine?”) and the assessment of the framework conditions of the planned course (9 questions, e.g. “What is the maximum workload per week for the part-time course?”) were collected. The survey was accompanied by an introductory definition of integrative oncology and an overview of the planned implementation. Three open questions allowed students to freely assess career prospects resulting from the course, interest in the topic within their own field and comments on the general circumstances. The answers were compiled and summarized for the evaluation. In total, 128 students participated (88.3% female, 10.9% male, 0.7% diverse). The students were on average 22 years old (M=22.31, SD=3.52) and came from the fields of nutritional sciences, social work, psychology, medicine, emergency services, communication, sports and nursing sciences, physiotherapy, occupational therapy and midwifery. Most students (74.0%) stated that their previous knowledge of integrative oncology and complementary medicine was rather poor, but rated the course as attractive both for themselves (56.1%) and generally for students of their respective study program (48.2%). Overall, 71.9% see the study program as a partial or complete alternative to the existing consecutive Master's programs offered in the respective disciplines (e.g. the M.Sc. in Nutritional Sciences). In terms of the general conditions, the interprofessional approach in particular was rated as attractive (M=3.5, SD=0.56), along with the described online format (M=3.1, SD=0.89), the admission requirements (M=3.1, SD=0.77) and the duration of the course (M=3.3, SD=0.66), which were rated on average as appropriate. Rated as particularly interesting were the topics of nutrition for cancer patients (M=3.6, SD=0.63), prevention (M=3.6, SD=0.58), follow-up care (M=3.4, SD=0.65) and physical activity with cancer (M=3.4, SD=0.73). The least interesting topic was spirituality (M=2.5, SD=0.98). The free text answers show that the topic is especially relevant for the field of nutritional sciences. For instance, 21 students state that nutrition is particularly relevant for the treatment of oncological diseases and serves as a health-promoting and therapy-supporting measure. In addition, 20 responses described the professional opportunity to specialize academically, resulting in better career opportunities in clinics. In terms of the general conditions, the requirement of one year of professional experience was seen as criticized, as the students (N=12) stated that getting a job at all as a newcomer is extremely difficult and therefore it would be hard to fulfill the requirement. 

The statements (N=15) were obtained by a guided interview via Zoom, transcribed and then corrected and confirmed by the interviewees. The questionnaire contained 3 thematic blocks: Previous contact with integrative oncology during university studies and at work, assessment of the demand and the target groups for the continuing education program, assessment of the attractiveness of the continuing education program. Students from the survey were contacted, as well as experts from the relevant disciplines who are already involved in integrative oncology and can therefore assess the field and the need for further education programs. In particular, the network of the PRIO (Prevention and Integrative Oncology) working group of the German Cancer Society was approached. As the largest and most established working group in the field of integrative oncology it has been offering certified courses in the field of integrative oncology since 2017. In order to ensure the anonymity of the respondents, no demographic information is provided. There were five statements from the field of medicine, two from nursing sciences, one from psychology, one from pharmacy, two from sports sciences and four students (two from nutrition, one from communication science and one from psychology). Overall, the consensus was that the course would be an attractive alternative and addition to existing courses for non-medical subjects, could expand the professional field und enable earlier practical involvement while maintaining the same high academic level (n=12). From the medical perspective, the continuing education program is particularly important with regard to the critical evidence-based analysis in the field of complementary medicine (n=4), as these topics undergo very diverse discussions and must take into account not only scientific, but also social and political aspects (e.g. in the field of homeopathy). The general conditions were consistently rated as manageable, especially for working people. However, all interviewees pointed out that the costs (in total: €12,900) could be an obstacle, especially for people from the nursing, nutrition and health sciences. Five people noted that the course content should also address networking and structuring in everyday clinical practice in order to be able to transfer the topics effectively into practice. In general, specializations in this subject area are highly requested (n=13), but so far hardly exist (n=10) and especially the scientific approach seems to meet a high demand (n=12). All respondents agree that the course closes a gap in demand (n=15).

## 4. Discussion and conclusion

The conception and implementation of a part-time continuing education course in the field of integrative oncology closes a gap in demand that has been confirmed from both a professional and student perspective. From the students' perspective, the topics of nutrition and physical activity were rated as particularly interesting and relevant for everyday care. All respondents felt that the general conditions were appropriate for part-time continuing education. A particular demand became apparent in the nutritional sciences, as specialized further training in the clinical field has been rather unusual to date and offers new career prospects. The academic level of the course, which focuses on a critical examination of the scientific evidence of the individual topics, was also rated as positive. One critical point to be noted is that the very tight timing of the separate implementation steps made it difficult to carry out a comprehensive and long-term demand assessment. Despite conducting the survey to the best of our ability, the results of the survey and statements are not generalizable due to the sample size and limitation to the Jena area (in the student sample) and do not allow to draw any universal conclusions about the demands or attitudes of the target groups. Due to the high proportion of women in the student survey, a distortion due to gender bias cannot be ruled out. Nevertheless, the statements from the practical perspective provided important information for the successful implementation of the course and therefore have a high qualitative value. The greatest criticism and skepticism arose with regard to tuition fees, which is understandable considering the different financial circumstances of the respective professions. However, since there is a self-financing obligation for continuing education course at German universities according to §6 of the “Thüringer Hochschulgesetz” *(Thuringian Higher Education Act)* [13], the scope for possible subsidization strategies is very small. Many similar continuing education programs also operate within a comparable financial framework. The course was approved by the “Thüringer Ministerium für Wirtschaft, Wissenschaft und Digitale Gesellschaft”* (Thuringian Ministry of Economics, Science and Digital Society)* in April 2023 and successfully started in the winter semester 2023-24. The fact that the first cohort has started despite the short-notice for applying to the course and the required tuition fee underlines the relevance of the course and the need for further education in the field of integrative oncology. The study program is included in the system accreditation of the Friedrich Schiller University and therefore has to be evaluated regularly. These future evaluation results will show whether the implementation of the course has been successful and what further optimizing measures can be taken.

## Author’s ORCID

Sarah Salomo: [0009-0006-3107-3597]

## Competing interests

The authors declare that they have no competing interests. 
